# Medical education and mental health during COVID-19: a survey across 9 countries

**DOI:** 10.5116/ijme.6209.10d6

**Published:** 2022-02-26

**Authors:** Daniel Michaeli, Gregory Keough, Francisco Perez-Dominguez, Francisca Polanco-Ilabaca, Fernanda Pinto-Toledo, Julia Michaeli, Sebastian Albers, Jadi Achiardi, Valeria Santana, Chiara Urnelli, Yoshihiro Sawaguchi, Perla Rodríguez, Mónica Maldonado, Zaheer Raffeeq, Otavio de Araujo Madeiros, Thomas Michaeli

**Affiliations:** 1 Department of Personalized Oncology, University Hospital Mannheim, Heidelberg University, Mannheim, Germany; 2 Department of Health Policy, London School of Economics and Political Sciences, London, United Kingdom; 3 Escuela de Salud Pública, Facultad de Medicina, Universidad de Chile, Santiago, Chile; 4 Department of Obstetrics and Gynecology, Asklepios-Clinic Hamburg-Altona, Asklepios Hospital Group, Hamburg, Germany; 5 Department of Orthopedic Surgery, ATOS Klinik Fleetinsel Hamburg, Hamburg, Germany; 6 Facultad de Medicina y Ciencias de la Salud, Universidad Militar Nueva Granada, Bogotá, Colombia; 7 Escuela Luis Razetti, Universidad Central de Venezuela, Caracas, Venezuela; 8 Facoltà di Medicina e Chirurgia, Università degli Studi del Piemonte Orientale, Novara, Italy; 9 Faculty of Medicine, Tokyo Medical and Dental University, Tokyo, Japan; 10 Facultad de Medicina, Universidad Panamericana, Ciudad de México, Mexico; 11 Facultad de Medicina, Universidad de Sevilla, Sevilla, Spain; 12 Humanitas University - Hunimed, Milan, Italy; 13 Faculdade de Ciências Médicas Santa Casa de São Paulo, São Paulo, Brazil; 14 Fifth Department of Medicine, University Hospital Mannheim, Heidelberg University, Mannheim, Germany

**Keywords:** COVID-19, mental health, medical student, depression, insomnia

## Abstract

**Objectives:**

To investigate students' experience with medical education alongside their mental and physical health since the onset of the COVID-19 pandemic across nine countries.

**Methods:**

A cross-sectional online survey was distributed by local collaborators to 2,280 medical students across 148 medical schools in Brazil, Chile, Colombia, Germany, Italy, Japan, Mexico, Spain, and Venezuela using non-probability convenience sampling from June 22 to July 24, 2020. Students answered questions regarding teaching, internet use, COVID-19, physical and mental well-being. A multivariate logistic regression examined factors associated with depressed mood, insomnia, and headache.

**Results:**

Academic teaching shifted to a virtual (67%, n=1,534) or hybrid environment (23%, n=531), whilst bedside teaching was suspended or cancelled (93%, n=2,120). Across all countries students were equally satisfied with the teaching modality, quantity, quality, and the evaluation system of in-person, hybrid, and online curricula. Negative changes in mental (40% (n=912) insomnia, 57% (n=1,300) emotional irritability, 47% (n=1,072) emotional instability, 41% (n=935) anhedonia, 40% (n=912) depressed mood) and physical (36% (n=821) headache, 57% (n=1,299) ocular tiredness, 49% (n=1,117) backache) health symptoms were frequently observed. Positive associations between the number of daily screen hours and depressed mood (adjusted odds ratio (AOR)=1.09, 95%CI: 1.05-1.12, p<.001), insomnia (AOR=1.08, 95%CI: 1.05-1.11, p<.001), and headache (AOR=1.11, 95%CI: 1.07-1.14, p<.001) were identified.

**Conclusions:**

Students' experience with digital and hybrid medical curricula was diverse during the pandemic. Education modality, quantity, and quality were positively evaluated. However, students' mental and physical health worsened. Besides bedside teaching, faculties ought to digitalize and strengthen social communities and extend support services for students.

## Introduction

Similar to previous outbreaks, the COVID-19 pandemic and its consequent policy responses disrupted social and professional life.^1^^-^^5^ While university hospitals focused on providing care to patients, medical education and students' well-being were deprioritized. Since the pandemic's onset medical schools unavoidably adjusted academic curricula in order to adhere to social distancing, safety, and health policies.^6^^,^^7^ Most schools shifted to online teaching with a reduction or even suspension of practical teaching.^6^^-^^8^ Practical exams, such as objective structured clinical examination (OSCE), were replaced with online, open-book exams.^9^^-^^11^ Consequently, students faced uncertainty surrounding prolonged studies, delayed graduation, and feared a cancellation of residency programmes.^11^^, ^^12^ Not only did this change complicate academic learning, but it also disrupted social peer networks of students.^7^^,^^13^ As well informed and educated future healthcare professionals, students were frequently mobilized and fast-tracked to support shortages in medical staff.^7^^, ^^9^^, ^^14^^-^^16^ Nevertheless, some authors embraced the long-overdue disruption of rigid education systems towards a hybrid, practical and/or virtual curriculum.^17^^-^^20^ Some specialties, e.g., otolaryngology or elective surgery, suffered disproportionally from immediate and future effects.^7^^,^^12^^,^^21^^-^^23^ In conclusion, the pandemic presented as an unexpected, disruptive external shock to medical schools and students. Theoretically, the resulting uncertainty and turmoil of academic curricula, future career prospects, and social networks may adversely affect mental health. However, the association of these academic and social changes triggered by the pandemic on mental health remain unclear.

Numerous studies revealed the pandemic's harmful impact on mental health.^2^^-^^5^^,^^24^ Proximity to high-risk areas, especially for medical personal and students, was frequently identified as a key risk factor for deteriorating mental well-being.^25^^-^^30^ Surveys in China, Vietnam, Australia, and Saudi Arabia previously investigated the prevalence and risk factors associated with mental health disorders among medical students during COVID-19. Depression was frequently associated with risk factors such as female sex, pessimistic thoughts, and anxiety disorders.^31^^, ^^32^ Anxiety and insomnia were significantly increased among graduate students with pessimistic thoughts and depression who lived in high-risk regions.^31^^,^^32^ Mental distress was even more prevalent and associated with direct COVID-19 infection, infected relative, alcohol consumption, smoking, female sex, low health literacy, and recent enrolment.^33^^,^^34^ However, present research investigating medical student's psychological health is limited in geographical scope and size.^31^^-^^37^

To the best of our knowledge, there is no study that thoroughly examines medical students' experience and well-being during the COVID-19 pandemic. Consequently, the present study fills this research gap by investigating and contrasting medical students' experiences during the COVID-19 pandemic across nine countries (Brazil, Chile, Colombia, Germany, Italy, Japan, Mexico, Spain, and Venezuela). The objective of this study is twofold:

•    First, we aimed to describe changes in medical education since the onset of the pandemic and to compare how well students perceived different teaching modalities (online, hybrid, in-person) during the pandemic.

•    Second, we aimed to examine changes in students' study habits, mental health, physical health, and substance use since the onset of the pandemic. Within this, we intended to identify factors associated with negative changes in depressed mood, insomnia, and headache.

## Methods

### Study design and participants

A cross-sectional study was conducted from June 22 to July 24, 2020. First, a multidisciplinary group composed of medical students, public health doctors, a mathematic engineer, and a psychologist designed a five-part online survey. The Spanish survey was translated into five different languages (English, German, Italian, Portuguese, Japanese) by official interpreters. Non-probability sampling using convenience sampling technique was employed to acquire 2,280 full survey responses from medical students.

### Demographic characteristics

The analysis includes 2,280 completed survey responses (completion rate: 79.7%) from Brazil (157), Chile (565), Colombia (260), Germany (550), Italy (169), Japan (150), Mexico (124), Spain (122), and Venezuela (183). 68% (n=1,545) identified as female, 31% (n=710) as male, and 1% (n=25) as other. The average medical student was 22.5 years old and enrolled in the third year of medical school. Across the entire sample, 16% (n=370) of students were involved in the care of COVID-19 patients. Medical students from Germany (36%, n=198) were more, while those from Japan (3%, n=5), Mexico (5%, n=6), Spain (2%, n=2), and Venezuela (8%, n=15) less frequently involved in the care of infected patients (χ²_(8, N=2,280)_=237.43, p<.001). Baseline characteristics are presented in Table 1.

All respondents acknowledge to be registered medical students. While medical education lasts six years in all surveyed countries, the curriculum's structure varies across nations. In Germany, Spain, Colombia, and Chile, the medical curriculum consists of two pre-clinical and four clinical years. In contrast, Japanese students are educated for four years on pre-clinical and two years on clinical subjects. In Italy and Venezuela, students go through three years of theoretical and three years of clinical education. The last year of medical school is mostly dedicated to clerkships and medical internships to gain practical experience. Nonetheless, even within countries, there are discrepancies of the employed medical curriculum across faculties. For instance, some faculties in Germany employ an integrated curriculum, e.g.,

**Table 1:** Baseline characteristics of the survey sample

Variable			No.	(%)
Gender				
	Male		710	(31)
	Female		1,545	(68)
	Other		25	(1)
Age, mean (y) ^a^			22.5	[22.4-22.7]
Year of study, mean (y) ^a^			3.61	[3.54-3.69]
Screen time, mean (h) ^a^			9.68	[9.54-9.82]
Teaching modality				
	No teaching		150	(7)
	In-person		65	(3)
	Hybrid		531	(23)
	Online		1,534	(67)
Academic duration				
	Unchanged		956	(42)
	Changed / postponed		1,324	(58)
No. academic evaluations				
	Unchanged		1,256	(55)
	Changed / postponed		1,024	(45)
Practical activities				
	Unchanged		160	(7)
	Reduced		826	(36)
	Suspended		1,294	(57)
Thought about postponing studies?				
	No		1,372	(60)
	Yes		908	(40)
Cared for COVID-19 patients?				
	No		1,910	(84)
	Yes		370	(16)
Country				
	Brazil		157	(7)
	Chile		565	(25)
	Colombia		260	(11)
	Germany		550	(24)
	Italy		169	(7)
	Japan		150	(7)
	Mexico		124	(5)
	Spain		122	(5)
	Venezuela		183	(8)
Overall sample (N)			2,280	(100)

a Brackets represent 95% confidence intervals

Medical Faculty Mannheim of Heidelberg University, whereas others follow traditional teaching structures. Across all surveyed countries, students generally commence medical school directly after high school. However, students can also take gap years or pursue a paramedic apprenticeship before starting medical education – ultimately impacting the age at which students enter school.

The study was approved by the "Ethical Committee in Human Beings of the University of Chile." The survey also followed Chilean law Nº 20.584 that regulates people's rights and duties regarding the actions linked to health care and respects the international norms of data protection by the Organization of American States. All participants gave written online consent before starting the survey. To ensure confidentiality, all responses were anonymized.

### Measures

The five-part survey consisted of 41 items lasting approximately ten minutes. The first part collected demographic characteristics regarding age, gender, year of study, country, university, and satisfaction with their internet connection. Satisfaction was measured on a scale from 1 to 5 (1: very satisfied, 2: satisfied, 3: indifferent, 4: unsatisfied, 5: very unsatisfied). The second part asked questions about the student's academic program, including changes in study modality, duration of the current year, evaluations, and practical activities. Participants were questioned about their current learning and study experience in the third part. Student's satisfaction with teaching modality, quantity, quality, the valuation system, academic burden, academic achievement, and the school's employed sanitary measures was measured on a scale from 1 to 5 (1: very satisfied, 2: satisfied, 3: indifferent, 4: unsatisfied, 5: very unsatisfied).

The fourth part investigated physical and mental health. Participants were asked about perceived changes in lifestyle habits (sleep, eating, weight), mental health (insomnia, emotional irritability, emotional instability, anhedonia, depressed mood), and physical health (headache, ocular tiredness, backache). For each outcome, students could report positive, negative, and neutral changes. Questions also addressed the use of substances and drugs (alcohol, tobacco, cannabis, other drugs, antidepressants, psychostimulants). For each item, students could select between decreased, increased, and unchanged consumption. The fifth part contained pandemic-related questions, e.g., engagement in the care for COVID-19 patients.

The survey was tested (n=50) in Chile, Colombia, Brazil, Mexico, Italy, Spain, Germany, and Japan to ensure validity and reliability. Students provided feedback on the survey's time, comprehensibility, user-friendly format, level of interest, and general suggestions. The final survey was adjusted according to pre-test results.

### Data collection and study setting

Medical student collaborators, who were enrolled students at medical faculties within the nine surveyed countries, distributed the survey in each location via institutional emails, student associations, and social media using the non-probability convenience sampling technique. A total of 148 medical schools across nine countries (Chile, Colombia, Brazil, Mexico, Italy, Spain, Germany, and Japan) were included in the final analysis. The cross-sectional survey was conducted from June 22, 2020, to July 24, 2020.

### Data analysis

Demographic characteristics and curricular changes were examined in absolute numbers and percentages. Students' satisfaction with in-person, hybrid and online teaching formats was compared using Chi-squared (χ²)-tests and visualized on a Likert-plot. The mean number of mental and physical health symptoms was compared across countries using χ²-tests and displayed on a world map. Changes in substance and drug use were displayed for each country. Multivariate logistic regressions explore the association of collected variables with depressed mood, insomnia, and headache. Adjusted odds ratios (AOR) and 95% confidence intervals (CI) are presented. Data analysis was performed with STATA SE Version 15.1.

## Results

### Curricula changes

In the entire sample, students reported that their faculties implemented the following teaching modalities: 7% (n=150) no teaching, 3% (n=65) in-person, 23% (n=531) hybrid, and 67% (n=1,534) online. Overall, 58% (n=1,324) reported a change / postponement in the academic duration and 45% (n=1,024) in the number of academic evaluations. 36% (n=826) reported a reduction and 57% (n=1,294) a suspension of practical activities in academic health facilities since the onset of the pandemic. All results varied across countries.

**Figure 1:**
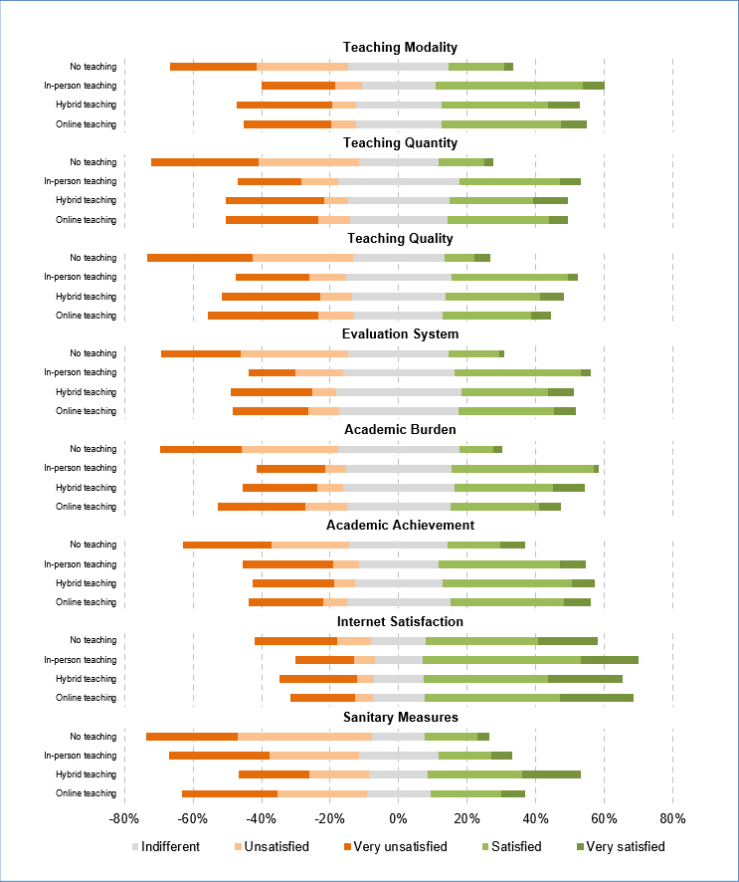
Medical students' satisfaction with in-person, hybrid, and online teaching formats

Students' satisfaction with the implemented teaching modalities varied significantly (Figure 1). Across all countries, students were equally satisfied with the teaching modality, quality, the evaluation system, and academic burden of in-person, hybrid, and online curricula. Students were more satisfied with the teaching quantity of in-person teaching (M=3.0, SD=1.1) compared to hybrid (M=3.1, SD=1.1) and online (M=3.2, SD=1.1) teaching (χ²_(8, N=2,130)_=22.44, p=.004). Students were generally pleased with their internet connection (M=2.5, SD=1.2). Respondents educated under a hybrid curriculum (M=2.9, SD=1.4) were more content with the local COVID-19 sanitary measures than in-person (M=3.5, SD=1.2) or online (M=3.5, SD=1.3) teaching (χ²_(8, N=2,130)_=76.58, p<.001). Across all dimensions, students were more unsatisfied with no teaching than other teaching modalities.

### Mental and physical health symptoms

In the entire sample, at least every fifth student experienced negative changes in lifestyle habits: 21% (n=479) sleeping, 29% (n=661) eating, 22% (n=502) weight. Even more frequently students witnessed undesirable shifts in mental health symptoms: 40% (n=912) insomnia, 57% (n=1,300) emotional irritability, and 47% (n=1,072) instability, 41% (n=935) anhedonia, and 40% (n=912) depressed mood. Similarly common were negative changes in physical health: 36% (n=821) headache, 57% (n=1,299) ocular tiredness, and 49% (n=1,117) backache.

**Figure 2:**
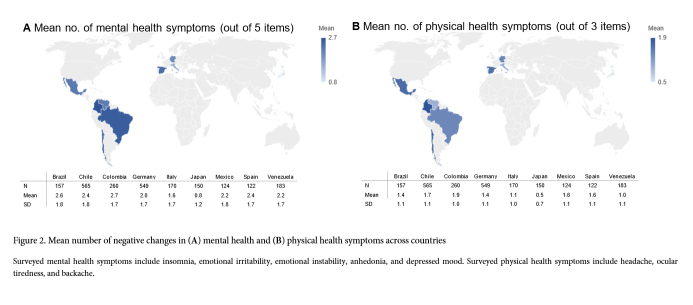
Mean number of negative changes in (A) mental health and (B) physical health symptoms across countries

On average students reported 2.2 (SD=1.8) mental and 1.4 (SD=1.1) physical health symptoms that were new or intensified since the onset of the pandemic. However, mental (χ²_(40, N=2,280)_=228.56, p<.001) and physical (χ²_(24, N=2,280)_=259.71, p<.001) health symptoms were unevenly distributed across countries (Figure 2). On the lower end, medical students in Japan only reported 0.8 (SD=1.2) mental and 0.5 (SD=0.7) physical health symptoms. In contrast, Colombian and Brazilian students displayed 2.7 (SD=1.7) and 2.6 (SD=1.8) mental health symptoms, respectively. Physical health symptoms were especially frequent in Colombia (M=1.9, SD=1.0), Chile (M=1.7, SD=1.1), Mexico (M=1.6, SD=1.1), and Spain (M=1.6, SD=1.1).

### Substance and drug use

**Figure 3:**
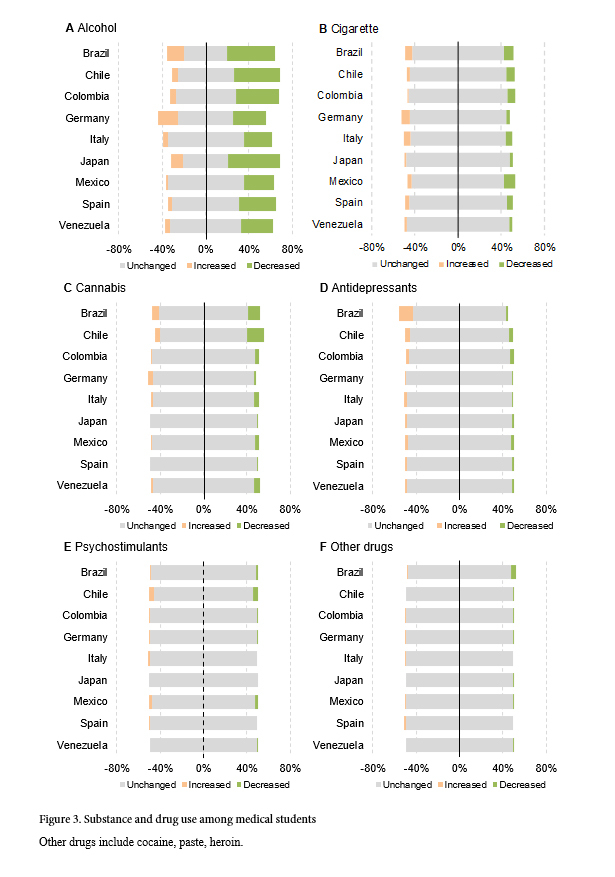
Substance and drug use among medical students

Across all countries, 36% (n=828) of students reported a reduction in their alcohol consumption, while 9% (n=210) reported an increase. However, regional differences exist as a surge in alcohol consumption was more frequent in Germany with 19% (n=102), Brazil with 16% (n=25), and Japan with 11% (n=16). Overall, smoking habits remained unchanged, given that 4% (n=96) of students increased and 5% (n=135) reduced their smoking. Similarly, 3% (n=71) of respondents increased their consumption of cannabis whereas 7% (n=151) decreased it. Three percent (n=77) of students increased their use of antidepressants, while 2% (n=48) diminished it. Notably is the surge of antidepressant use in Brazil with 13% (n=20) and Chile with 5% (n=27). The use of psychostimulants and other drugs remained largely unchanged across all countries (Figure 3).

### Variables associated with depressed mood, insomnia, and headache

The multivariate regression (Table 2) suggests that female relative to male students were at higher risk to develop negative changes in depressed mood (AOR=1.60, p<.001), insomnia (AOR=1.40, p<.001), and headache (AOR=2.36, p<.001). Medical students in their last years of education compared to beginners were at a lower risk of developing mental or physical health symptoms. Students that participated in the care of COVID-19 patients were not at an increased risk to develop any of the three health symptoms. Students experiencing negative changes in their studying habits since the onset of the pandemic were at higher risk of erosions in mental and physical health – depressed mood (AOR=2.10, p<.001), insomnia (AOR=1.80, p<.001), and headache (AOR=1.46, p<.001). Similarly, students with changed/postponed curricula durations were more susceptible to negative changes in depressed mood (AOR=2.13, p<.001), insomnia (AOR=1.64, p<.001), and headache (AOR=1.34, p<.001) symptoms.

Changes in the teaching modality – online vs hybrid or in-person – were not significantly associated with health outcomes. Students that experienced changes in the academic duration were more susceptible to mental and physical health symptoms – depressed mood (AOR=1.15, p=.218), insomnia (AOR=1.38, p=.005), and headache (AOR=1.28, p=.040). Reductions in the number of practical courses and exams were not associated with a significant change in mental or physical health symptoms. Medical students who were dissatisfied with their internet connection were more likely to develop depressed mood (AOR=1.14, p=.002), insomnia (AOR=1.07, p=.115), and headache (AOR=1.18, p<.001). Accordingly, positive associations between the number of daily screen hours

and all three outcomes were identified – depressed mood (AOR=1.09, p<.001), insomnia (AOR=1.08, p<.001), and headache (AOR=1.11, p<.001).

Respondents stating an increased alcohol consumption were at a higher risk of developing a depressed mood (AOR=1.42, p=.038) and insomnia (AOR=1.41, p=.042). Smoking was significantly correlated with new onsets and intensification of insomnia (AOR=1.92, p=.004), whereas the consumption of cannabis was correlated to headaches (AOR=1.75, p=.040). Consumption of antidepressants was correlated to depressed mood (AOR=3.32, p<.001) and headache (AOR=2.03, p=.011).

**Table 2:** Multivariate logistic regression of factors associated with depressed mood, insomnia, and headache

Variable	Depressed Mood ^a^				Insomnia ^a^				Headache ^a^		
	AOR	[95% CI]	p-value		AOR	[95% CI]	p-value		AOR	[95% CI]	p-value
Gender											
Male	1	[Reference]			1.00	[Reference]			1.00	[Reference]	
Female	1.60	[1.30,1.96]	p<.001		1.40	[1.14,1.71]	p<.001		2.36	[1.90,2.92]	p<.001
Other	2.23	[0.87,5.74]	p=.096		2.57	[0.93,7.13]	p=.069		1.76	[0.50,6.23]	p=.383
Year of study	0.91	[0.86,0.96]	p<.001		0.96	[0.91,1.02]	p=.156		0.88	[0.83,0.93]	p<.001
The COVID-19 pandemic negatively	1.42	[1.15,1.76]	p<.001		1.27	[1.03,1.56]	p=.024		1.15	[0.94,1.42]	p=.184
impacts your quality as a doctor?											
Satisfaction with local	1.07	[0.98,1.18]	p=.149		1.03	[0.94,1.13]	p=.491		1.13	[1.03,1.25]	p=.010
COVID-19 sanitary measures ^b^											
Online teaching	NA	NA	NA		1.11	[0.82,1.50]	p=.494		1.04	[0.75,1.45]	p=.808
Medical school delayed semester	1.15	[0.92,1.45]	p=.218		1.38	[1.10,1.72]	p=.005		1.28	[1.01,1.61]	p=.040
Reduced no. of exams	0.91	[0.74,1.11]	p=.344		NA	NA	NA		NA	NA	NA
Thought about delaying studies?	2.13	[1.73,2.62]	p<.001		1.64	[1.34,2.00]	p<.001		1.34	[1.09,1.65]	p=.005
Negative change in study habits?	2.10	[1.73,2.55]	p<.001		1.80	[1.49,2.19]	p<.001		1.46	[1.20,1.78]	p<.001
Internet satisfaction ^b^	1.14	[1.05,1.23]	p=.002		1.07	[0.98,1.16]	p=.115		1.18	[1.09,1.28]	p<.001
Daily screen time (h)	1.09	[1.05,1.12]	p<.001		1.08	[1.05,1.11]	p<.001		1.11	[1.07,1.14]	p<.001
Increased alcohol consumption	1.42	[1.02,1.98]	p=.038		1.41	[1.01,1.95]	p=.042		NA	NA	NA
Increased cigarette consumption	1.37	[0.85,2.20]	p=.191		1.92	[1.23,2.99]	p=.004		NA	NA	NA
Increased cannabis consumption	NA	NA	NA		NA	NA	NA		1.75	[1.03,3.00]	p=.040
Increased antidepressant use	3.32	[1.83,6.02]	p<.001		1.39	[0.81,2.41]	p=.235		2.03	[1.18,3.51]	p=.011
Increased psychostimulant use	1.84	[0.85,3.95]	p=.121		1.84	[0.88,3.87]	p=.108		1.21	[0.58,2.50]	p=.613
Increased use of other drugs ^c^	NA	NA	NA		1.70	[0.33,8.76]	p=.525		1.70	[0.31,9.38]	p=.545
Country											
Brazil	1.00	[Reference]			1.00	[Reference]			1.00	[Reference]	
Chile	0.74	[0.48,1.13]	p=.162		1.02	[0.68,1.52]	p=.932		1.89	[1.23,2.91]	p=.004
Colombia	0.87	[0.54,1.42]	p=.585		1.47	[0.93,2.34]	p=.101		2.29	[1.39,3.76]	p<.001
Germany	2.00	[1.24,3.23]	p=.005		1.06	[0.67,1.66]	p=.809		2.04	[1.25,3.33]	p=.005
Italy	1.33	[0.75,2.34]	p=.325		1.18	[0.67,2.05]	p=.571		1.55	[0.85,2.79]	p=.150
Japan	0.53	[0.29,0.98]	p=.042		0.43	[0.23,0.80]	p=.008		0.50	[0.24,1.01]	p=.055
Mexico	0.79	[0.46,1.35]	p=.387		2.01	[1.20,3.38]	p=.008		1.47	[0.84,2.57]	p=.178
Spain	1.25	[0.70,2.22]	p=.446		1.70	[1.00,2.88]	p=.049		2.76	[1.59,4.79]	p<.001
Venezuela	0.95	[0.57,1.58]	p=.841		0.75	[0.46,1.24]	p=.265		0.75	[0.44,1.30]	p=.305

a New onset or intensification of depressed mood, insomnia, or headache since the onset of the pandemic. b Satisfaction of internet connection and local sanitary measures was assessed with a 5-point Likert scale from 1 ("very dissatisfied") to 5 ("very satisfied"). c Other drugs include cocaine, paste, heroin.

## Discussion

This cross-sectional survey investigated students' experience with medical school curricula alongside their mental and physical health during the COVID-10 pandemic across nine countries. We observed that medical faculties shifted academic teaching to a virtual (67%, n=1,534) or hybrid environment (23%, n=531), whilst bedside teaching was suspended or cancelled (93%, n=2,120). However, our findings indicate a mixed experience with virtual teaching. Education modality, quality, the evaluation system, and academic burden were evaluated similarly to in-person and hybrid teaching. Although we observed high levels of mental and physical distress, alcohol consumption declined whilst cigarette and cannabis use remained largely unchanged. Students' consecutively increased screen time was positively correlated to negative changes in depressed mood, insomnia, and headache.

### Medical education and COVID-19

Medical hospitals were not only confronted with more infected COVID-19 patients but also had to maintain medical teaching. The medical curriculum had to rapidly incorporate new public policies whilst also equipping students with crucial hands-on skills and introducing them to clinical routine.

The present study found drastic shifts in the academic curricula of medical schools. Similar to previous case reports and general expectations, classes were predominantly virtual or hybrid.^6^^-^^8^^, ^^38^ Differences across countries may partially be explained by the differential impact of the pandemic among countries and varying digital infrastructures. Online teaching provides students with similar theoretical knowledge as in-person curricula and also promotes self-paced learning.^39^ On the other hand, laptops, phones, and other technology devices were found to be a source of distraction, are not equally accessible across all countries and sample groups, and seldom teach social skills – which are essential for the work of doctors.^40^ In our survey, 45% of students reported worsening of their study habits alongside frequent physical symptoms, which could adversely impact their concentration and learning abilities. Nonetheless, the observed changes in study habits and physical health symptoms could be equally caused by the transition to a virtual curriculum as well as public policies that limit social life. Medical schools could organize classes to prepare students for remote learning requiring adapted study habits – in the long run, this may strengthen resilience and foster successful academic adaptation during a pandemic.

Students' satisfaction with medical curricula across the surveyed countries were divergent. There was no difference in the students' satisfaction regarding teaching modality, quality, and quantity of in-person, hybrid, and online teaching. Students under an online teaching environment were less satisfied with the overall academic burden. Notably, across all examined dimensions, "no teaching" was evaluated worse than any of the other teaching formats. This result underlines that students want to continue their education and seek guidance from their university during the uncertain pandemic. Additionally, two-thirds of students across all countries were afraid that the direct and indirect effects of the pandemic would negatively impact their quality as a physician. In other words, certain parts of the medical curriculum ought not to be digitized. Bedside teaching, doctor-patient anamnesis, practical examinations, and laboratory classes are essential for the comprehensive training of emerging physicians.^39^^,^^41^ Medical schools must decide until when and to what extent the current virtual/hybrid medical curriculum will persist after the pandemic subsides. The pandemic might allow a transformation in medical education with the active participation of students in curricular innovation. Students should learn new technologies, such as telehealth, during their regular curricula. Students' overall satisfaction could be increased by establishing decentralized interactions in safe environments with patients to limit the risk of transmission of the virus.

### Medical students' mental and physical health

Coherent with previous studies, our results support the association between pandemics and surges in physical and mental distress.^3^^,^^42^^-^^45^ Relative to medical student reference groups, we observed high levels of mental and physical health symptoms. This detected psychological pain could be caused by social distancing and quarantining measures that limit social life and be augmented by the novel digital/hybrid medical teaching. Previous studies found that both policies are associated with increased psychological distress.^2^^-^^5^ Physical and mental distress might be improved by the introduction of more advanced technologies, such as virtual clinical experiences. Students need to acquire tools to combat stressors within a social support environment. Resilience during COVID-19 is higher with coping strategies when students perceive a high level of social and emotional support and thereby ameliorate their mental health.^46^ Further studies are necessary to analyze to what magnitude medical students' deteriorated well-being persists beyond the pandemic.

As teaching transitioned to a virtual/hybrid curriculum, students inevitably had to spend more time in front of a screen every day. However, our results reveal a high correlation between daily screen time and depressed mood, insomnia, and headaches. This is in line with several studies confirming the relationship between display use and psychological distress as well as physical illnesses.^47^^,^^48^ A higher use of electronic devices was especially linked to depression, anxiety, and lower emotional stability rates.^47^^,^^48^ Furthermore, increased internet usage was identified as a risk factor to develop an addiction for patients with underlying psychological comorbidity to develop an addiction.^49^ Further studies reveal that screen time is a risk factor to develop a metabolic syndrome in a dose-dependent manner independent of physical activity.^49^ In contrast, a recent review underlines that regular physical activity has beneficial effects on depression and anxiety during the COVID-19 pandemic.^50^ Consequently, universities should facilitate and promote physical activities by a variety of means, e.g., offering online activity courses, enhancing access to sport facilities, or providing fitness community apps. Staying physically active during the COVID-19 pandemic would contribute to the attenuation of the side effects caused by the outbreak on mental health after the pandemic subsides. Consequently, online curricula should allow for sufficient breaks to facilitate screen absence. More strikingly, based on the findings of a meta-analysis, social isolation and loneliness have a comparable effect on mortality, such as with well-established risk factors for mortality.^51^ Taken together, there might be long-term consequences on the medical students' health. The transition to an online curriculum may accelerate existing inequities within a country and across nations. Access to a good internet connection is a prerequisite for online education, yet 25% of students reported a poor connection. A poor connection does not only hinder widespread adoption of virtual teaching, but is also a source of distraction, cultivates students' frustration, and inhibits learning success.^52^ Furthermore, a relatively poor internet connection might be a proxy for a lower socioeconomic status. Medical faculties must be aware of the direct and indirect consequences of novel policies on their students.

The current year's academic duration changed or was uncertain for more than half of the surveyed students. In accordance with expectations, this change and uncertainty were strongly and significantly associated with depressed mood, insomnia, and headache. Students may frequently perceive this external insecurity with worries about their short-term academic future, but also long-term career prospects.^11^^,^^12^ In line with expectations, practical teaching activities were frequently reduced or suspended. The absence of practical education coupled with hesitation encircling digital teaching formats could have intensified the observed emotional distress.^39^^, ^^41^ Medical universities might react to students' worsened mental health by offering easily accessible psychological counselling and interventions. Moreover, the interaction with teachers, classmates, friends, and other social peers should be strengthened.

Results also suggest that mental health symptoms were more prevalent in the early stages of medical school compared to later stages. In countries such as Germany, Italy, or Spain, the pre-clinical stage is known to be especially work-intensive and stressful for students, which could rationalize this finding. Alternatively, more mature students have already built a stronger social network at their university and could thereby possess more coping mechanisms to combat external shocks such as the COVID-19 pandemic. As a result, medical universities could introduce mentors for freshmen to provide social and specialized support.

Increased alcohol consumption was significantly associated with negative changes in depressed mood and insomnia. Such associations are commonly observed in previous literature.^34^^,^^53^ Based on studies investigating alcohol consumption after the SARS outbreak in 2003, these results must be reviewed very carefully. Short-term increases in alcohol consumption were maintained in the general public and among healthcare workers after the 2003 outbreak.^53^ Similarly, results show that students displaying psychological disorders were prescribed or self-medicated more frequently with antidepressants. Antidepressants might not be sufficient to treat depressive symptoms. Generally, physician consultation is necessary to evaluate and diagnose a mental illness. After that, a combination of pharmacotherapy, a strong network of social support, and psychotherapy are necessary to combat underlying causes of mental illnesses in the long run. Therefore, virtual social peer networks and mentoring groups can help to increase and maintain social capital.^19^ However, the findings reveal that 36% (n=821) of the sample reported a reduction in their alcohol consumption. This might be explained by increased access barriers due to reduced opening hours and frequent lockdown policies.

### Limitations

Limitations of this study are present. First, online survey biases impact our results. Self-selection bias of internet users and the gender unbalanced sample (68% female) limit the generalizability of results. Students could have been dishonest about reporting substance use, especially if such substances are illegal in their country. Second, mental health variables were not assessed with validated survey tools. Mental health outcomes were self-reported and, therefore, not clinically verified. Third, causality cannot be inferred given the cross-sectional survey design. Moreover, variations in the structure of medical education and licensing exams limit the comparability and generalizability of results across countries. In addition, participation in online surveys is limited by the respondents' access to the internet. As a result, results might over-report access to online/hybrid classes. Finally, findings only provide insight into health outcomes until July 24, 2020. Further studies are necessary to explore the long-term association between academic/societal changes and mental health status due to COVID-19.

## Conclusions

The COVID-19 pandemic not only disrupted social and professional life but also drastically impacted academic teaching for medical students. Adding to existing evidence, this study confirms the high rates of mental and physical health burdens faced by medical students across all nine countries. Psychological distress was especially frequent for students with a poor internet connection. A prolonged academic year, increased alcohol and antidepressants consumption, and a higher screen time was associated with mental and physical symptoms. Policymakers and schools should be aware of these burdens, as non-traditional academic curricula may extend beyond 2021. The findings of the study implicate that students' mental health and medical education might be improved by ensuring high-speed internet connection. Moreover, daily screen time should be limited despite online education. In addition, access barriers allowing online contact to affected peers and mental health coaches should be lowered. However, even after the pandemic subsides and academic life returns to normality, the presented severe changes in mental health may persist. Therefore, targeted psychological interventions are crucial to meet students' health during and beyond the pandemic. Panel studies are required to examine the casual long-term implications of COVID-19 on mental health.

### Acknowledgements

The authors would like to thank Mr Juan Pedro Ross and Mr Francisco Lopez for collaborating in the initial design of the study and all medical students who participated in this survey.

### Conflict of Interest

The authors declare that they have no conflict of interest.
